# Supplementary material for: Impact and Implementation of an Early Years Fundamental Motor Skills Intervention for Children 4–5 Years
Source: Children (Basel). 2024 Apr 1;11(4):416. doi: 10.3390/children11040416 (PMC11048878; doi:10.3390/children11040416)
Supplement: Supplementary file 1 [file children-11-00416-s001.zip › children-2893926-supplementary.pdf]

Class: EYFS	Date:
Topic: Lesson 2 Method of travel	

What we are learning today (WALT):

- Developing movements using different methods of travel
- Exploring ways of travelling using different parts of the body

Student Numbers: Male: _____ Female: _____ SEN: _____ G&T: _____

Times	Starter activities:	Differentiation:
	**"Here, There, Everywhere"** - (Running "Here" = To me)<br>(Running "There" = Wherever I point)<br>(Running "everywhere" = I chase & tag you)<br>**"Musical statues"** - (Walk, run, jog, dance, skip - freeze when music stops)<br><br>**Learning activities:**<br>**"Sausage sizzle"** - (Log rolls along the mats)<br>**"Sammy snail"** -(Lie on Tummy, bean bag on back, commando crawl, don't lose your house)<br>**"Crab Grab"** - (Bean bags all around the room, pick up bean bags using feet, put in to the basket.)<br>**"Jumping jelly"** - (Jump feet together, count jumps least jumps to win)<br>**"2 Forward, 1 back"** - (Jump feet together, 2 forwards and 1 back, from 1 side of the room to the other)<br><br>**Plenary activities:**<br>**Plenary Activity (Cool down):**<br>**"Stretch" -** (Arms, fingers, backs & legs)	

Evaluation:	Resources Needed:
	Mats x6, Bean bags, Baskets, Music

Class:	Date:
Topic: Lesson 3 Balance and Linking Actions	

What we are learning today (WALT):

Student Numbers: Male: _____ Female: _____ SEN: _____ G&T: _____

Times		Differentiation:
	**Starter activities:**<br><br>**"Traffic lights"** - (Green - go fast, Amber - slow down, Red - Stop)<br>**"Dotty Spot"** - (Follow instructions by coach, ie: stand in front, stand behind, one foot on the floor, one hand etc.)<br><br>**Learning activities:**<br><br>**"Lame dog**" - (On all fours, hands and knees, follow instructions ie: left arm, right arm, left leg, right leg etc. Hold each item up for 5 seconds)<br>**"Buzz bridge"** - (Walk in pairs, one on all fours, hands and feet with hands in a hoop, other child pass the hoop along body to feet without touching the body)<br>**"Step on, off, over"**- (Using a bench, children step on, off and over carefully!)<br>**"High Jinx jump"** - (Using a bench, jump onto a mat- strtech, star, truck, quarter turn, half turn, full turn)<br>**"Jump to the beat"** - (Using music, marching, jumping jacks with hands on hips, grapevine, kicker twister & rocker)<br>**Plenary activities:**<br><br>**"Sleeping Donkey" -** (Child on fours, hand and knees. Close eyes and wait)	

Evaluation:	Resources Needed:<br>Mats x 3, hoops, spots, bench and music
